# Chromosomal assignment of canine *THADA *gene to CFA 10q25

**DOI:** 10.1186/1755-8166-1-11

**Published:** 2008-06-03

**Authors:** Jan T Soller, Claudia Beuing, Hugo Murua Escobar, Susanne Winkler, Nicola Reimann-Berg, Norbert Drieschner, Gaudenz Dolf, Claude Schelling, Ingo Nolte, Jörn Bullerdiek

**Affiliations:** 1 Centre for Human Genetics, University of Bremen, Leobener Straße ZHG, 28359 Bremen, Germany; 2 Small Animal Clinic and Research Cluster of Excellence "REBIRTH", University of Veterinary Medicine Hanover, Bischofsholer Damm 15, 30173 Hannover, Germany; 3 Institute of Genetics, Vetsuisse Faculty, University of Berne, Bremgartenstrasse 109a, PO Box 8466, 3001 Bern, Switzerland; 4 Department of Animal Science, Swiss Federal Institute of Technology Zurich, Vetsuisse-Faculty Zurich, University of Zurich, Winterthurerstrasse 204, 8057 Zürich, Switzerland

## Abstract

**Background:**

Chromosomal translocations affecting the chromosome 2p21 cluster in a 450 kb breakpoint region are frequently observed in human benign thyroid adenomas. *THADA *(thyroid adenoma associated) was identified as the affected gene within this breakpoint region. In contrast to man tumours of the thyroid gland of dogs (*Canis lupus familiaris*) constitute mainly as follicular cell carcinomas, with malignant thyroid tumours being more frequent than benign thyroid adenomas. In order to elucidate if the *THADA *gene is also a target of chromosomal rearrangements in thyroid adenomas of the dog we have physically mapped the canine *THADA *gene to canine chromosome 10.

A PCR was established to screen a canine genome library for a BAC clone containing the gene sequence of canine *THADA*. Further PCR reactions were done using the identified BAC clone as a template in order to verify the corresponding PCR product by sequencing.

Canine whole blood was incubated with colcemid in order to arrest the cultured cells in metaphases. The verified BAC DNA was digoxigenin labeled and used as a probe in fluorescence *in situ *hybridization (FISH). Ten well spread metaphases were examined indicating a signal on canine chromosome 10 on both chromatids. A detailed fine mapping was performed indicating the canine *THADA *gene locus on the q-arm of chromosome 10.

**Results:**

The canine *THADA *gene locus was mapped on chromosome 10q25. Our mapping results obtained in this study following the previously described nomenclature for the canine karyotype.

**Conclusion:**

We analysed whether the *THADA *gene locus is a hotspot of canine chromosomal rearrangements in canine neoplastic lesions of the thyroid and in addition might play a role as a candidate gene for a possible malignant transformation of canine thyroid adenomas. Although the available cytogenetic data of canine thyroid adenomas are still insufficient the chromosomal region to which the canine *THADA *has been mapped seems to be no hotspot of chromosomal aberrations seen in canine thyroid adenomas.

## Background

In human thyroid adenomas chromosomal translocations involving the regions 19q13 and 2p21 have frequently been described [1,2]. Chromosomal aberrations showing 2p21 rearrangements belong to the most common abnormalities in benign epithelial tumours with an observed frequency of 10% [3]. Recently, a gene named *THADA *(thyroid adenoma associated) [GenBank: NM_022065] which is directly affected by this cytogenetic rearrangement, could be identified within the 2p21 breakpoint region [3].

In terms of animal cancer models, the dog has lately been attracting significant interest due to the fact that the malignancies of humans and dogs show various similarities [4]. Among the striking arguments for the dog as an animal model for man are spontaneous appearance of the tumours, comparable histological variance, similar cancer types and similar biological behaviour of the observed neoplasias, including metastasis [5-7].

In man the majority of tumours affecting the thyroid gland are benign, whereas in dogs the situation is quite different. Thyroid carcinomas are rare in the human population, the overall incidence is <1% [8,9]. A total of 1.2% of all canine tumours affects the thyroid gland with thyroid carcinomas occurring more frequently than adenomas [9,10]. Carcinomas of follicular origin are the most common form of canine thyroid neoplasias [11].

In order to elucidate whether human genes which are involved in the pathogenesis of benign thyroid adenomas could play a role as orthologous candidate genes for a possible malignant transformation of thyroid tumours in dogs, we have mapped the canine *THADA *gene and analysed if the canine gene locus is involved in cytogenetic rearrangements.

## Methods

### BAC library screening

A PCR reaction for PCR-based screening of the *Canis familiaris *DogBAC library [12] (Institute of Animal Genetics, Nutrition and Housing, University of Berne, Berne, Switzerland) for a BAC clone containing *THADA *was established using canine genomic DNA derived from blood. The primers T1: 5'GCATTTTTCGATTGTCATAAC'3 and T2: 5'TCAGCCAAAACTAGATAACAC'3 were designed using the predicted canine *THADA *gene sequence of canine chromosome 10, [GeneBank: NC_006592]. PCR parameters were: 95°C for 5 min, followed by 35 cycles of 95°C 30 sec, 55°C 30 sec, 72°C 30 sec, and a final elongation of 72°C for 10 min. The corresponding 601 bp PCR product was cloned into the pGEM-T Easy vector system (Promega) and verified by sequencing. The obtained canine sequence contained the 97 bp of exon 2 with a 92% similarity to human exon 2 of *THADA *[GenBank: NM_022065]. The sequence was submitted to the NCBI database [GenBank DQ836130]. The DNA contigs and alignments were done with Lasergene software (DNAstar, Madison, USA) and various sequences from the NCBI database [GeneBank: AC_000045, NM_022065].

For verification and secondary screening a semi-nested PCR reaction was established, using the T1 primer and a nested primer T1B (5'TCAGTACTATTGGCATTTGGAG'3) generating a 147 bp amplicon. A positive BAC clone (DogBAC library ID S011P24K05RE) was identified and verified by PCR. The obtained PCR products were cloned into the pGEM-T Easy vector system (Promega) and verified by sequencing. The verified clone was used as probe for following FISH experiments.

### Slide Preparation

1 ml of canine whole blood was incubated for 72 h in Chromosome Medium B (Biochrom). Subsequently, colcemide (0.1 μg/ml) (Biochrom) was added for 2 hours. The cells were centrifuged at 135 × g for 10 min and incubated for 20 min in 0.05 M KCl. Finally, the cells were fixed overnight with methanol/glacial acetic acid. This suspension was dropped on ice-cold slides and dried for at least 7 days at 37°C. The chromosomes were stained by GTG banding for karyotype description. Prior to use in FISH investigations, the slides were destained with 70% ethanol.

### Fluorescence in situ hybridization

BAC-DNA was digoxigenin labeled (Dig-Nick-Translation-Kit, Roche). The hybridization mixture contained 200 ng probe, 40 ng ssDNA, 600 ng sonicated dog DNA, 2 × SSC, 2 × SSPE, 50% formamide and 10% dextran sulfate. 50 μl of this mixture were applied to each slide and the cover slips were sealed with rubber cement. Probe and chromosomes were denatured at 75°C on an Eppendorf thermocycler gradient, using the in situ adapter. Afterwards, the slides were incubated in a moist chamber at 37°C over night. Cover slips were carefully removed and the slides were incubated in 0.1 × SSC at 61°C and 1 × PBS at RT. Slides were then covered with 100 μl NFDM for 20 min. at 37°C in a moist chamber. For signal detection 100 μl NFDM containing 3 μg of anti-digoxigenin-rhodamine, fab fragments (Roche) were added to each slide and again incubated for 20 min at 37°C in a moist chamber, followed by washes with 1 × PBS, 3 × 3 min at RT. Slides were air dried before chromosome staining was performed with 25 μl of Vectashield mounting medium with DAPI (Vector Laboratories).

**Figure 1:**
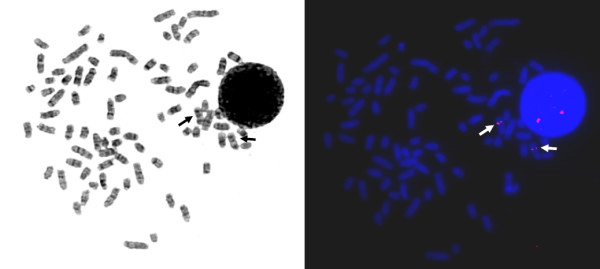
**FISH mapping of canine THADA**. Metaphase spread after fluorescence *in situ *hybridisation with signals on CFA 10q25 (right) of both chromosomes, the same metaphase after GTG-banding (left).

Ten well spread metaphases were examined indicating a signal on canine chromosome 10 on both chromatids of both chromosomes of canine chromosome 10 (Fig. 1). The determination of chromosomes followed the nomenclature of the canine karyotype as described previously [13].

## Results and Discussion

In humans 10% of all chromosomal rearrangements in benign thyroid tumours involve a 450 kb breakpoint region on 2p21 [14]. These 2p21 rearrangements are the most commonly detected cytogenetic aberrations in benign thyroid adenomas. Within the mentioned breakpoint region the *THADA *gene was identified as a target gene for translocation events. *THADA *transcripts fused downstream to ectopic sequences of human chromosome 3 and 7 have been detected in thyroid adenoma cell lines S325/TSV40 and S533/TSV40 [3] (Centre for Human Genetics, University of Bremen, Bremen, Germany). The loss of parts of the *THADA *coding sequence is proposed to play a major role in the pathogenesis of these lesions affecting the thyroid gland due to the truncation of the gene and its deduced protein sequence [3,15]. Rippe et al. (2003) proposed an involvement of THADA in a death receptor pathway and characterized the human *THADA*. The mRNA of *THADA *contains 6090 bp (ORF 5862 bp) and 38 exons and encodes a hypothetical protein of 1954 amino acids.

The UCSC genome browser on Dog May 2005 [16] assembly showed the canine *THADA *chromosomal location on CFA 10 at region 48,883,266 to 49,209,580 and the gene encompasses a sequence of 326,315 bp. The genome browser also described that the canine *THADA *region is highly conserved between the homologous gene locations of man, mouse and rat. Particularly the *THADA *region seems to be more evolutionarily conserved between man and dog than in rodents. In detail NCBI Blast analyses [17] were performed in order to show similarities between various mammalian and avian *THADA *nucleotide sequences from the NCBI databases. The similarities of the canine *THADA *[GenBank: EF222204] coding sequence (CDS) to the CDS of other species vary between human CDS [GenBank: NM_022065] 87%, mouse CDS 80% [GenBank: EF222207], rat predicted CDS 80% [GenBank XM_001060686.1] and chicken 69% [Genbank EF222206]. Respectively the similarities of the canine proposed THADA protein sequence to the deduced protein sequence of other species vary between human protein 83% [GenBank: NP_071348], mouse protein 75% [GenBank: ABQ10601], rat predicted protein 76% [XP_233829] and chicken protein 59% [GenBank: NP_001103529]. Also all deducted mammalian and avian THADA proposed protein sequence have in common one highly conserved domain termed COG5543 [CDD: 35102], the clusters of orthologous groups protein motif with yet unknown function, identified by the NCBI Conserved Domain Database (CDD) [18].

The mapping of *THADA *to canine chromosome 10 shows that the chromosomal region to which the canine *THADA *has been mapped is not a hotspot of chromosomal aberrations seen in canine thyroid adenomas. Previous case reports of canine thyroid adenomas showed either a trisomy of chromosome 18 as a sole cytogenetic abnormality [19], or a rather complex karyotype of chromosomal fusions [20]. However, the available cytogenetic data of canine thyroid adenomas are still insufficient.

In 1999, reciprocal chromosome painting probes were established for a comparative chromosome map between human, red fox and dog showing the hybridisation pattern of canine probes onto human chromosomes [21,22]. Corresponding to the data obtained by the painting probes conserved syntheny exists between canine chromosome 10 and the human chromosomes 2, 12, and 22. Our mapping result obtained in this study is in accordance with the described homology of the canine and human chromosomes allowing a fine mapping of the *THADA *gene locus on canine chromosome 10q25 (Fig 1.) which corresponds to the 2p21 region of the p-arm of human chromosome 2.

## Conclusion

In dogs malignant thyroid carcinomas occur more often than adenomas but interestingly no cytogenetic reports of canine thyroid carcinomas have been published until now (NCBI, Pubmed 2008). In order to elucidate whether canine *THADA *could be a candidate gene for a possible malignant transformation of canine thyroid adenomas further cytogenetic studies of the tumours could be of significant value.

## Competing interests

The authors declare that they have no competing interests.

## Authors' contributions

JTS and CB carried out the molecular genetic studies, established the PCR condition, performed the *in silico *analyses and drafted the manuscript, SW and ND carried out the FISH, SW and NRB determined the gene locus and performed the fine-mapping following the nomenclature of the canine karyotype, GD and CS screened the DogBAC library for a BAC clone containing gene of interest, HME, IN and JB conceived the study, participated in the experimental design and coordination, and helped to draft the manuscript. All authors read and approved the final manuscript.
